# Comparative analysis of vaginal microbiota sampling using 16S rRNA gene analysis

**DOI:** 10.1371/journal.pone.0181477

**Published:** 2017-07-19

**Authors:** Seppo Virtanen, Ilkka Kalliala, Pekka Nieminen, Anne Salonen

**Affiliations:** 1 Obstetrics and Gynecology, University of Helsinki and Helsinki University Hospital, Helsinki, Finland; 2 Institute of Reproductive and Developmental Biology, Department of Surgery and Cancer, Imperial College London, London, United Kingdom; 3 Immunobiology Research Programme, Department of Bacteriology and Immunology, University of Helsinki, Helsinki, Finland; Fred Hutchinson Cancer Research Center, UNITED STATES

## Abstract

**Background:**

Molecular methods such as next-generation sequencing are actively being employed to characterize the vaginal microbiota in health and disease. Previous studies have focused on characterizing the biological variation in the microbiota, and less is known about how factors related to sampling contribute to the results. Our aim was to investigate the impact of a sampling device and anatomical sampling site on the quantitative and qualitative outcomes relevant for vaginal microbiota research. We sampled 10 Finnish women representing diverse clinical characteristics with flocked swabs, the Evalyn® self-sampling device, sterile plastic spatulas and a cervical brush that were used to collect samples from fornix, vaginal wall and cervix. Samples were compared on DNA and protein yield, bacterial load, and microbiota diversity and species composition based on Illumina MiSeq sequencing of the 16S rRNA gene. We quantified the relative contributions of sampling variables versus intrinsic variables in the overall microbiota variation, and evaluated the microbiota profiles using several commonly employed metrics such as alpha and beta diversity as well as abundance of major bacterial genera and species.

**Results:**

The total DNA yield was strongly dependent on the sampling device and to a lesser extent on the anatomical site of sampling. The sampling strategy did not affect the protein yield or the bacterial load. All tested sampling methods produced highly comparable microbiota profiles based on MiSeq sequencing. The sampling method explained only 2% (p-value = 0.89) of the overall microbiota variation, markedly surpassed by intrinsic factors such as clinical status (microscopy for bacterial vaginosis 53%, p = 0.0001), bleeding (19%, p = 0.0001), and the variation between subjects (11%, p-value 0.0001).

**Conclusions:**

The results indicate that different sampling strategies yield comparable vaginal microbiota composition and diversity. Hence, past and future vaginal microbiota studies employing different sampling strategies should be comparable in the absence of other technical confounders. The Evalyn® self-sampling device performed equally well compared to samples taken by a clinician, and hence offers a good-quality microbiota sample without the need for a gynecological examination. The amount of collected sample as well as the DNA and protein yield varied across the sampling techniques, which may have practical implications for study design.

## Introduction

Along with the general trend in the human microbiome field, high-throughput molecular methods, predominantly next-generation sequencing (NGS), are widely used to provide untargeted identification and relative quantification of vaginal bacteria, including species that cannot be cultured. Alterations in the vaginal microbiota and host-microbe interactions have been implicated in a number of gynecological and obstetric conditions, such as bacterial vaginosis [1], cervical cancer [2,3], infertility [4], preterm birth [5] and sexually transmitted infections (STI) [6]. Apart from disease, the vaginal microbiota is affected by various intrinsic factors such as menstrual cycle [7], pregnancy [8], ethnicity [9] and menopause [10] and by external factors such as smoking [11]. Evidently, understanding the normal variation of the vaginal microbiota and the role of different host-associated factors is imperative for interpretation of the data and identifying diagnostic microbiota signatures. The contribution of technical variation introduced by specimen collection and storage, DNA extraction, and sequencing should also be known and optimally minimized to enable cross-comparison of the different studies, and to unmask the underlying biological variation.

Previously, a study comparing the performance of different sample collection methods for vaginal soluble proteins [12] found that endocervical swabs and lavage samples collected higher cytokine and antimicrobial protein concentration compared to vaginal swabs. Also, the impact of the anatomical sampling site on the vaginal microbiota composition has been previously analyzed in two culture-independent studies with contradicting results: A clone-library based shallow sequencing assessment of the microbiota from eight women [13] suggested that both the sampling site and the device had substantial impact on the resultant microbiota, and due to the high variation of the replicate samples within individual, a single swab sample is not representative of the overall vaginal microbiota. On the other hand, the Human Microbiome Project sampled 113 women from vaginal introitus, mid-vagina and posterior fornix, and concluded based on pyrosequencing that the taxonomic profiles between the sites were essentially similar [14,15].

Self-sampling of vaginal content has been validated for the detection of individual pathogens such as high-risk types of human papilloma virus (hrHPV) [16] and *Chlamydia trachomatis* [17], as well as for targeted analysis of certain commensal bacteria [18]. Self-sampling was also shown to be suitable for community-wide microbiota analysis based on comparison of self- versus physician-collected samples from mid-vagina [19]. However, there are no previous data regarding the comparison of self-collected samples to possible spatial or sampling device-specific variation of microbiota in the vagina. Other potential sources of technical variation in vaginal microbiota sampling have been studied before, e.g. sample storage conditions [20] and bacterial DNA extraction [21]. However, this is the first study to compare different vaginal sites and sampling devices with NGS.

Non-biased, reproducible and validated strategies are needed to collect vaginal samples for microbiota analysis. Therefore, we have here employed partial 16S rRNA gene deep sequencing (NGS) to test whether the bacterial diversity and composition differs between commonly used vaginal sampling devices and different vaginal sites. We also wanted to test how representative the microbiota derived from a self-sampling device is compared to samples taken by a clinician from spatially specified sites, in order to estimate the usefulness of the self-sampling device for large-scale microbiota studies.

## Materials and methods

### Subjects and sampling

We sampled the vaginal microbiota of 10 premenopausal women at the Department of Obstetrics and Gynecology, Helsinki University Hospital Colposcopy Clinic in Southern Finland with seven different sampling methods, summing up to total of 70 samples. All subjects were recruited and sampled in April 2014. As each woman served as their own control regarding the different sampling strategies, we did not aim for homogenous population but instead included women of varying age, colposcopy result, use of contraceptives and presence of blood in the samples (Table A in S1 Appendix). Patients were not pregnant (self-reported) and they had more than 48 hours since last intercourse, except patient number 8 who had 36 hours. A Pap smear was taken from all patients and the presence of bacterial vaginosis (BV) was evaluated from smears with microscopy, using clue-cells and Nugent score features as diagnostic criteria [22]. We also recorded smoking status, contraceptive use, last menstrual period and recent antibiotic use.

**Fig 1:**
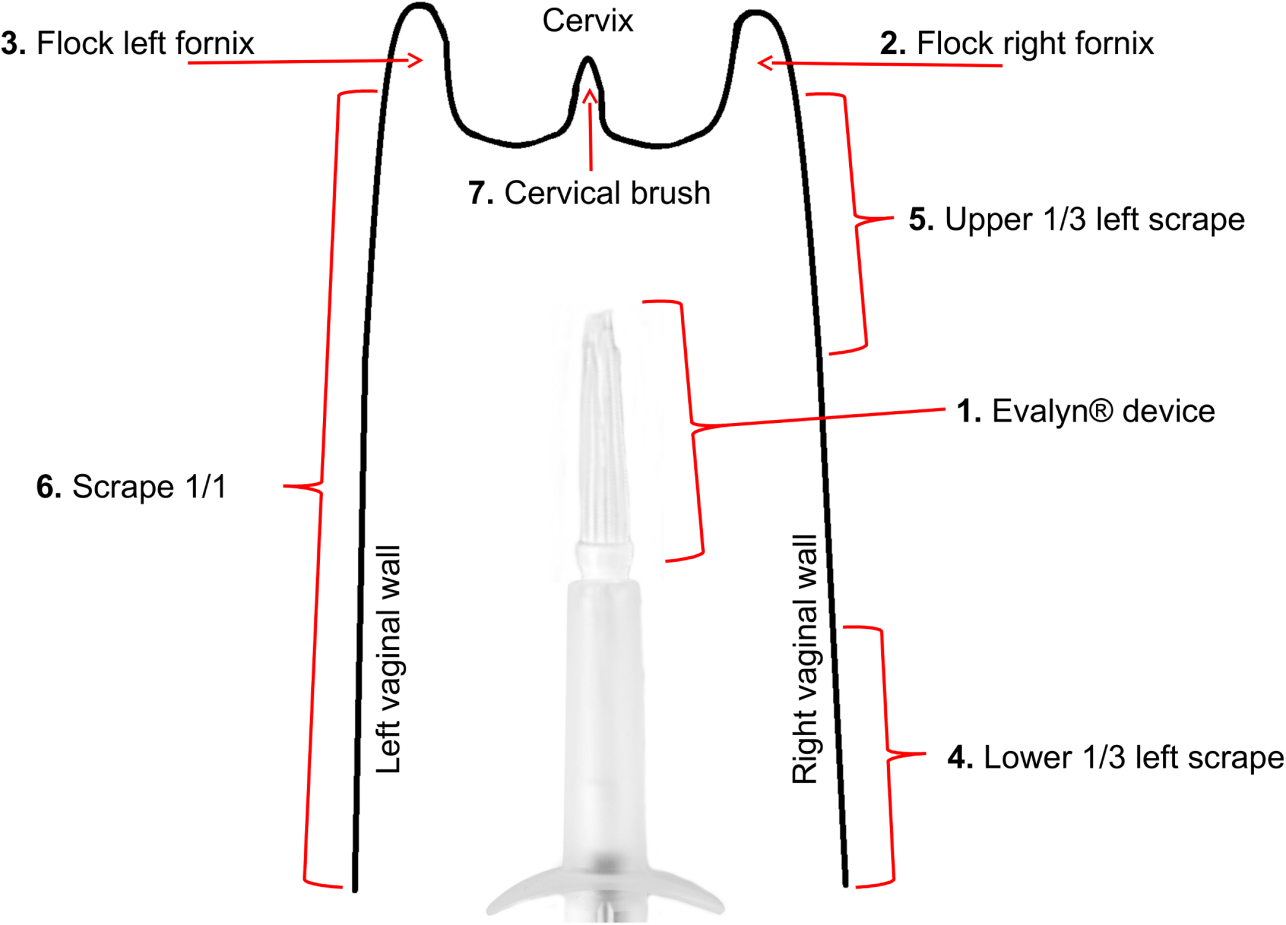
Sampling locations and devices. Schematic representation of vagina with arrows labeled with sampling devices and pointing to sampling locations. The locations are numbered according to the sampling order.

We took samples from six vaginal locations with four different sampling devices as specified in Fig 1. A Flocked swab (Floqswab regular, Copan, USA) was chosen for ease of use and offers high sample elution. A cervical brush (Cytobrush Plus, Medscand, USA) is already routinely used in colposcopy and offers accurate sampling from a specific site. The Evalyn® self-sampling device (Rovers, Netherlands) developed for hrHPV-testing was included to address the quality of self-collected microbiota samples. The Evalyn® device has a brush that is inside a protective plastic tube during insertion, and hence does not collect material from vaginal introitus or the lowest parts of vagina. After insertion, the brush comes out of the tube and rotates when a plunger at the end of the device is pushed and rotated. The tube has small wings as stoppers so that the depth of the insertion is about 2 cm (see Fig 1). We chose this device for self-sampling to minimize skin contamination and to collect microbiota from specified and reproducible depth in vagina. Scrape samples taken from the lower and upper third as well as from the entire vaginal wall with plastic spatulas (sterile PS-plastic, Aylesbury-shape) were used as a reference to estimate the sampling depth, possible skin or vulvar contamination of self-sampling, and to gather possible adherent bacteria from the vaginal wall. All samples were fresh frozen in dry 2 ml Eppendorf tubes except the left fornix flocked swabs, which were frozen in 1 ml saline to test the potential effect of storage buffer (sample 3. in Fig 1.). The Eppendorf tubes were frozen in a container filled with dry ice in the examination room and transported to -20C freezer by the end of the day. The samples were processed for analysis within 2–10 days from collection. All sampling devices were sterile except the cervical brush. The samples, except for the self-taken sample, were collected by three experienced physicians right after insertion of speculum. The speculum was lubricated with small amount of sterile saline if needed.

### Sample processing

#### DNA extraction

We used a modification of Repeated Bead Beating (RBB) method that efficiently extracts bacterial community DNA based on comparative analysis of human fecal samples [23]. All frozen samples were thawed on ice and 1 ml of sterile ice-cold PBS was added, except for the left fornix flocked swabs that were stored in saline. Before the removal of the sampling device with sterile forceps the samples were vortexed for 30 secs to dislodge the bacteria and briefly spun down to avoid spills when opening the tube. An aliquot of the clarified supernatant was taken for the measurement of soluble protein content. The rest of the sample, including both the mucous pellet and the supernatant, was moved to 2 ml screw-cap tube and an equal volume of lysis buffer (500 mM NaCl, 50 mM Tris-HCl (pH 8.0), 50 mM EDTA, 4% SDS) was added. To break to cells, samples were bead beaten using a Precellys 24 high-throughput tissue homogenizer (Bertin Technologies, France) with 0.1 mm zirconium-silica beads (Biospec Products, Bartlesville, OK, USA) for 2 x 30 sec at 5500 rpm. DNA from the lysates was purified using QIAamp DNA Minikit (Qiagen, Hilden, Germany) without the lysis step according to the manufacturer’s instructions. The samples were eluted in 200 μl of kit elution buffer.

#### DNA and protein quantification

Nanodrop spectrophotometer (NanoDrop® Technologies, Wilmington, DE, USA) was used to measure DNA and protein content at 260 nm and 280 nm, respectively. The DNA and protein yields were calculated per sample as if the whole sample was used for these analyses. 0.8% agarose gels electrophoresis was used for the quantification and qualification of the DNA extracts.

#### qPCR

To quantify the amount of bacterial DNA in the samples, triplicate qPCR amplifications were performed using iCycler—IQ instrument (BioRad Laboratories, USA) and 1x HOT FIREPol® EvaGreen® qPCR Mix Plus (Solis Biodyne, Estonia) in a volume of 25 μl. The thermal cycling conditions included an initial DNA denaturation step at 95°C for 10 min followed by 40 cycles of denaturation at 95°C for 20 s, annealing at 56°C for 20 s, extension at 72°C for 30 s, and an additional incubation step at 88°C for 30 s to collect the fluorescent data. Bacterial DNA was quantified by amplifying 0.5 ng aliquots of each DNA extract with universal bacterial primers targeting the 16S rRNA gene [24]. The standard curves ranging from 10^2^ to 10^7^ copies were constructed using the full-length amplicons of 16S rRNA gene of *Bifidobacterium longum* to convert the threshold cycle (Ct) values into 16S rRNA gene copy numbers per ng of template DNA.

#### 16S rRNA gene amplicon sequencing

Illumina MiSeq paired-end sequencing of the hypervariable V1-V3 regions of the 16S rRNA gene with primers pA-forward AGAGTTTGATCMTGGCTCAG and pD'-reverse GTATTACCGCGGCTGCTG was performed at the Institute of Biotechnology, DNA Sequencing and Genomics Laboratory, University of Helsinki, Helsinki, Finland. The PCR primers contained 18-mer overhangs added to the 5’ ends resulting to amplicon size of 556 nt [25]. Replicate PCR products were pooled and purified with Agencourt AMPure XP magnetic beads (Agencourt Bioscience, USA) and subjected to a second PCR round with barcoded forward primers and a reverse primer, both of which attached to the respective 18-mer overhang sequences from the primers of the first PCR amplification. Phusion polymerase (Thermo Fisher Scientific, USA) with HF buffer and 2.5% DMSO were used. Cycling conditions for both PCR reactions consisted of an initial denaturation at 98°C for 30 s, followed by 15 cycles at 98°C for 10 s, 65°C for 30 s, and 72°C for 10 s, and then a final extension for 5 min. The quality control of libraries was performed with Bioanalyzer 2100 (Agilent, USA). We excluded 10 samples that had very low DNA yields (<2.5 ng/ul) or were otherwise considered to be of insufficient quality and quantity for sequencing. Summary of the sequenced 60 samples is in Table B in S1 Appendix. The sequences have been deposited at European Nucleotide Archive, ENA accession code: PRJEB21386.

#### Preprocessing of reads

**Fig 2:**
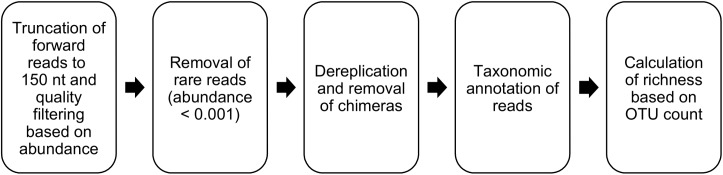
Pre-processing pipeline. MiSeq sequence pre-processing workflow with R-package mare [27].

MiSeq reads were processed in R [26] with package mare (Microbiota Analysis in R Easily) [27] as shown in Fig 2. The default parameters of mare, such as quality filtering based on read abundance rather than quality scores, use of only forward reads instead of the merged reads, and the truncation of sequences to 150 nt to include only the region of highest quality, were applied as they maximized the similarity between the expected and observed microbial richness and composition in mock communities [28]. The composition of mock communities used in parameter optimization has been described elsewhere [29]. The forward reads were truncated to length of 150 bases with mare’s “ProcessReads” command. We used default settings for minimum quality score (2) and maximum expected errors (1). Reads with abundance below 0.0001 were removed. Chimera removal and dereplication of the reads was done using USEARCH8 [30]. Truncated, filtered and dereplicated reads were then annotated using curated vaginal 16S V1-3 reference database from Fettweis et al. [31] to get species level classification with “TaxonomicTable” function of the mare package. Richness and diversity (inverse Simpson [32]) were estimated after clustering the reads to operational taxonomic units (OTUs) using USEARCH8.

### Statistical data analysis

The MiSeq data analysis was done without rarefaction or transformations utilizing statistical and visualization tools included in the mare-package [27]. To control for the effect of the variable read count between the samples, the number of reads was used as an offset in all statistical models. Basic statistical inference in mare is based on generalized linear models with negative binomial distribution utilizing glm.nb function of the R package MASS [33]. Community dissimilarity was estimated with principal coordinates analysis (PCoA) using Bray-Curtis dissimilarity as the distance measure. Bray-Curtis dissimilarity is 0 for identical samples and 1 for communities that do not share any species [34]. PCoA was calculated with the capscale function of R package vegan and Bray-Curtis dissimilarities with function vegdist of the same package [35]. Continuous variables in the PCoA-space were interpolated with gstat function of the gstat package in R [36]. To calculate the relative contribution of different variables in the microbiota variation, permutational multivariate analysis of variance using distance matrices was performed with adonis function in the vegan package. Taxonomic alpha-diversity was estimated as the number of observed OTUs and by the inverse Simpson’s diversity index. For univariate data (DNA yield, protein content, qPCR results, Bray-Curtis dissimilarity, richness, diversity) the differences between the samples was determined by repeated measures ANOVA in combination with Tukey’s post-hoc tests. We compared the relative abundances of the bacterial species collected with different methods with generalized linear mixed models within “GroupTest” function of the mare package using subject as the random factor. This function uses the glmmADMB package (generalized linear mixed models built on AD Model Builder) of R software on background and assumes negative binomial distribution of abundance (for the command and source code see S2 Appendix) [37,38]. P-values for taxon-specific differences were collected for false discovery rate (FDR; Benjamini–Hochberg [39]) with function p.adjust of the stats package in R [26].

## Results

We sampled 10 women at six different vaginal sites with four different devices to optimize and validate vaginal microbiota sampling for molecular studies. The comparison was based on quantitative and qualitative assessment, focusing on the microbiota profiles generated by sequencing of the hypervariable V1-V3 region of the 16S rRNA gene.

### Protein content

**Fig 3:**
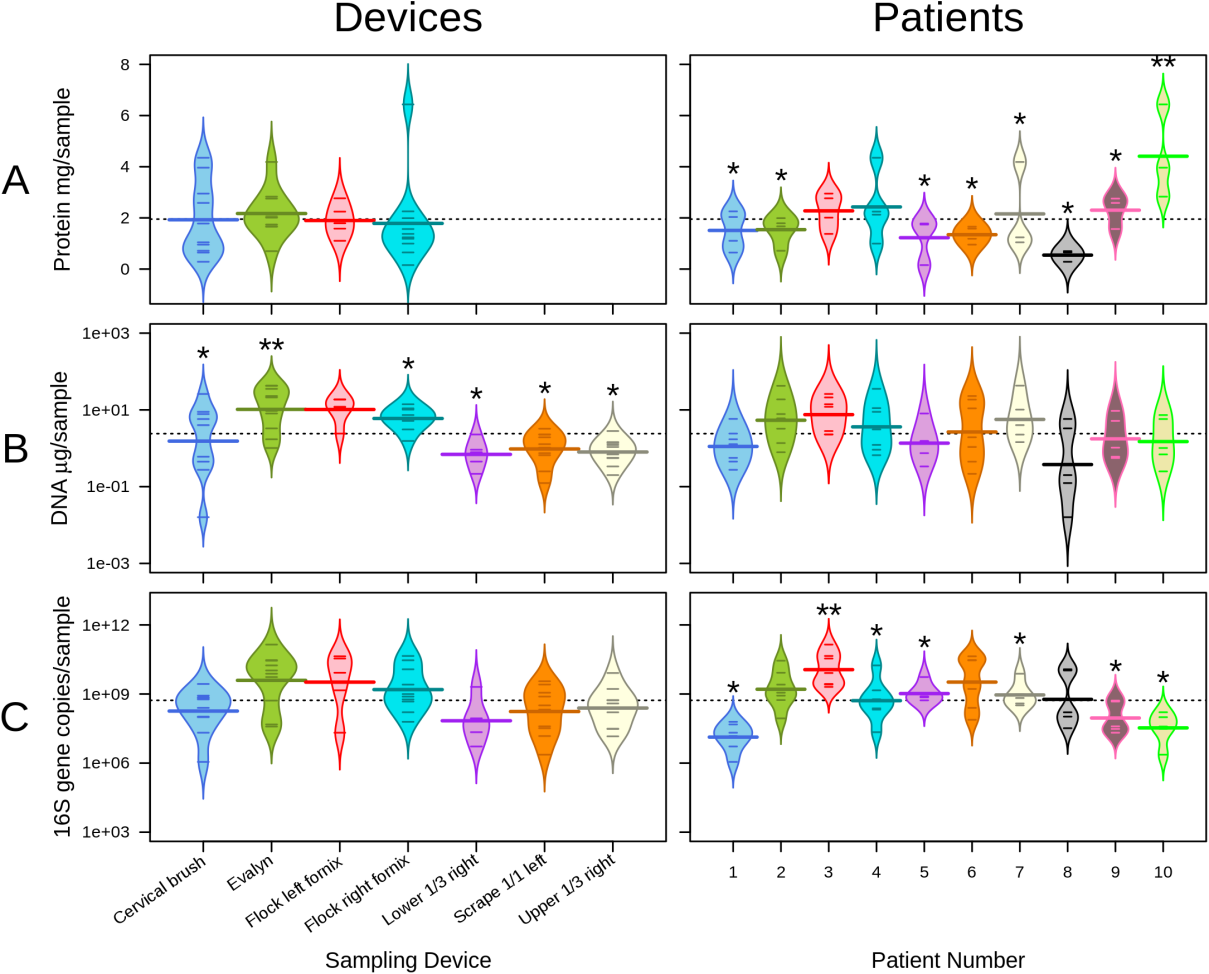
Protein and DNA results for different devices and patients. Protein yield (row A), DNA yield (row B) and total bacteria measured by qPCR (row C). The individual lines depict mean and the overall dotted line the group mean. The left columns show values according to the sampling methods (technical variation) and the and right according to the patients (biological variation). Protein yield was not measured from scrape samples due to small sample volume. Mean values not sharing the same number of asterisks signify samples with statistically significant (p < 0.05) pair-wise differences.

Protein content was measured for the flocked swabs, the self-taken sample and the cervical brush, because in contrast to the scrape-samples, those were thought to collect sufficiently material for the analysis of soluble immunomarkers and other proteins. All the tested devices collected roughly 2 mg of protein based on the Nanodrop measurement. There were no statistically significant differences in protein yield between the devices or vaginal sites, but there were significant, up to 8-fold, differences in protein yield between the patients (Fig 3A). Bleeding or the clinical status including cytological diagnosis and presence or absence of bacterial vaginosis did not have significant effect on the protein yield (Fig A-A in S1 Appendix).

### DNA yield and purity

The DNA yield varied with different sampling sites and devices. The mean DNA yields per sampling device varied up to 31-fold, the actual yields ranging from 0.16 ng to 42 μg per sample (Fig 3B). The mean DNA yield per device thus ranged from 596 ng to 19 μg. The highest DNA yield was obtained with self-sampling brush, followed by flock swabs, cervical brush and scrapes. The DNA yield of the self-taken sample was significantly higher than that of scrapes, cervical brush samples or right fornix flocked swab samples (all p-values < 0.05). Pair-wise comparison between the other devices did not yield significant differences. Bleeding or BV-status did not have effect on DNA yield (Fig A-B in S1 Appendix).

To assess the purity of different DNA preps we compared their A260/A280 and A260/A230 ratios from the Nanodrop measurement. The median of A260/A280 ratio was good (1.8–2.0), except for the scrape samples it varied from 2.4 to 4.8. As all samples were treated with RNAse during the DNA extractions, the higher A260/A280 ratio of scrape samples probably reflects the limitations of the Nanodrop performance with very low concentration samples (median 2.2–5.8 ng/μl DNA) or vulnerability to extraction artifacts/errors for the same reason. The A260/A230 ratio was also good (median 1.3–1.8) in all samples other than the scrapes (≤ 0.7), assumingly for the same reasons as above.

### Quantification of bacteria

To specifically quantify bacterial DNA in the different extracts, we used bacteria-specific qPCR assay and calculated the results as the number of 16S rRNA genes per ng of template DNA (ng^-1^ DNA) in order to normalize for the high device-driven variation in the absolute DNA yields. We then multiplied the number of 16S rRNA copies per ng with the DNA yield (corrected for the protein aliquot) to get the number of copies per sample. The mean amount of bacterial DNA varied 74 -fold between the methods and 648-fold between the patients. There were no statistical differences in the amount of bacteria collected across the tested devices and locations (Fig 3C), and no correlation between the DNA yield and bacterial load (Spearman r = 0.24, p = 0.07). Bleeding did not affect the bacterial content, but BV was associated with significantly higher bacterial counts compared to other clinical groups based on microscopy (Fig A-C in S1 Appendix).

### Composition and diversity analysis of the vaginal microbiota

#### Overview of the vaginal microbiota composition

**Fig 4:**
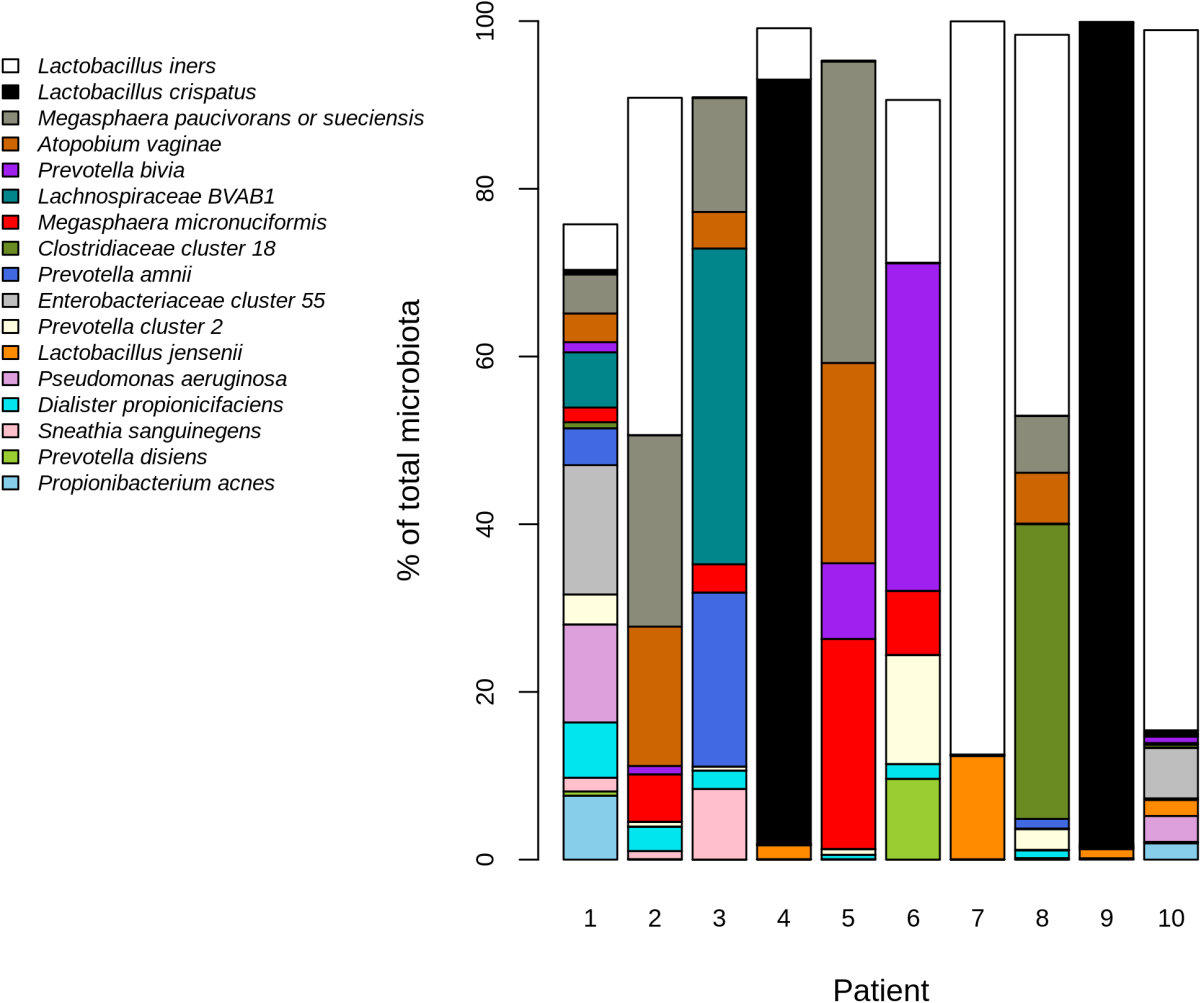
Abundant microbiota in the study subjects. The plot shows the most abundant bacteria (mean relative abundance > 0.01) per patient, representing mean abundance derived from all the sampling methods. Patients 3,5,6,8 had bacterial vaginosis according to Pap smear at the time of sampling. Patient 1 gave birth 2 months before sampling and post-partum atrophy was seen in Pap smear. Area not covered by the bars represents taxa below the abundance filter.

Illumina MiSeq sequencing was used to determine the impact of sampling devices and anatomical sites on the vaginal bacterial community composition. After preprocessing, we had an average of 79 500 high-quality sequences per sample (range 43 000–107 000). Due to the excess number of reads, there was no association between the read count and microbiota richness (Fig C in S1 Appendix). In the studied 10 Finnish asymptomatic women, we detected altogether 45 bacterial species. Most abundant bacteria were *Lactobacillus iners*, *Lactobacillus crispatus*, *Megasphera paucivorans/sueciensis*, *Prevotella bivia* and *Atopobium vaginae*. The variation of microbial composition was high between the individuals, six out of 10 women having *Lactobacillus*-dominated microbiota and the rest had *Enterobacter*, *Lachnospiraceae* BVAB1, *Prevotella* or *Megasphera* dominated community (Fig 4). Four patients had *L*. *iners* dominated (Community State Type III; CST III) and two *L*. *crispatus* dominated bacteria (CST I) [40]. We did not detect *Gardnerella vaginalis* in any of the samples, despite the fact that nine patients had abnormal bacterial findings suggestive of bacterial vaginosis in the Pap smear. The lack of *Gardnerella* sequences is likely reflecting PCR bias as the used V1 forward primer does not capture *Bifidobacteriales* including *G*. *vaginalis* [41].

#### Comparison of bacterial communities across sampling devices and sites

**Fig 5:**
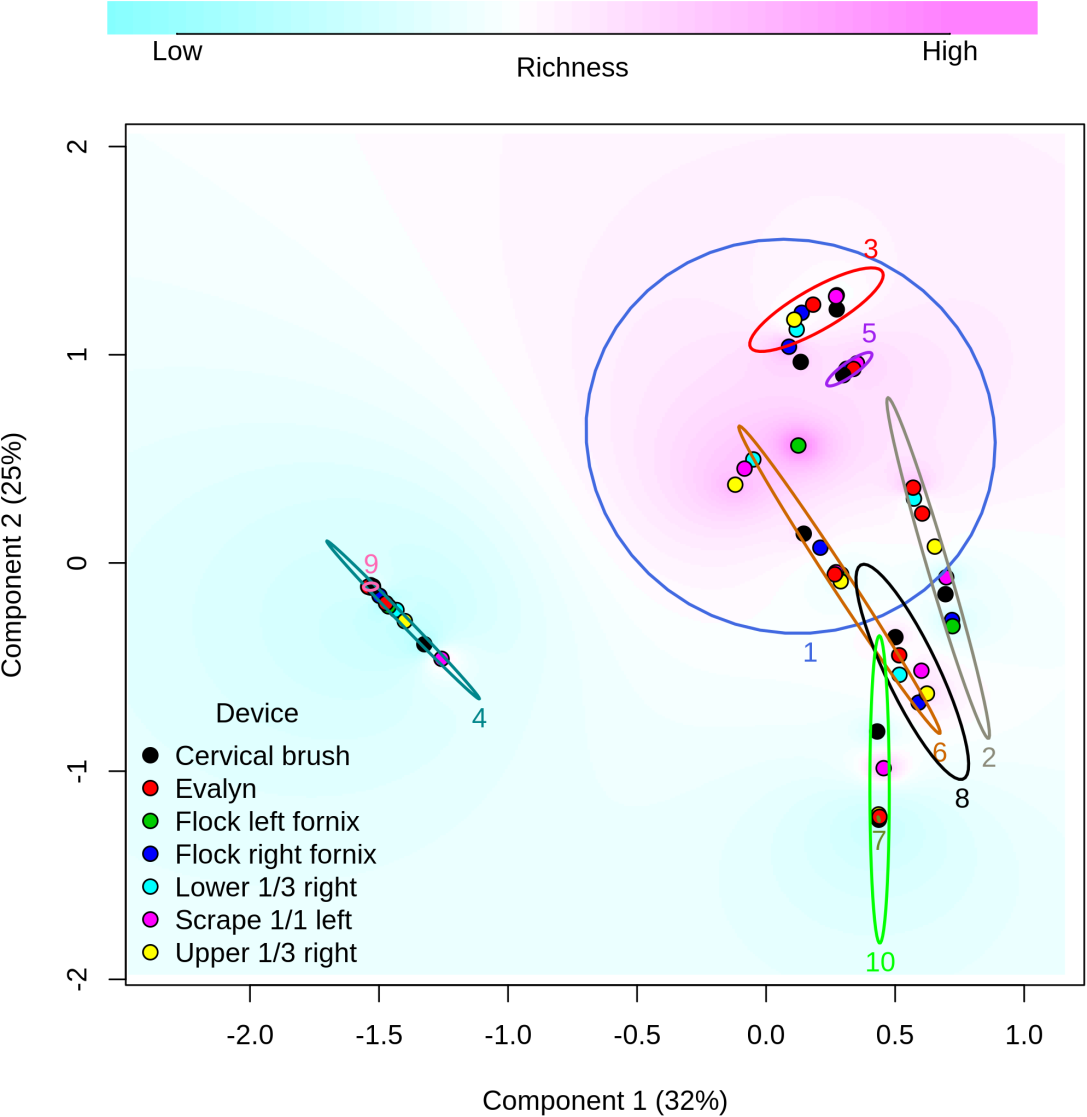
Principal coordinates analysis (PCoA) plot based on Bray-Curtis dissimilarity. Samples are colored by the sampling method, which explained only 2% of the microbiota variation (p = 0.89). Ellipses are drawn around every patients’ samples and numbered accordingly (patient; explaining 11%, p-value 0.0001). Component 1 explains 32% of variation and is dominated by *L*. *crispatus*, + *L*. *iners* and *Ureaplasma*. Component 2 explains 25% and is dominated by *L*. *iners*, + *Prevotella* and -*Eggerthella*.

Our focus was to determine the impact of sampling devices and sites on the overall community composition and diversity of the vaginal microbiota. Principal coordinates analysis (PCoA) based on Bray-Curtis dissimilarity of all the samples revealed that the vaginal microbial communities clustered according to the patient (Fig 5.). Permutational multivariate ANOVA, with Bray-Curtis dissimilarities, was used to assess the proportion of variation in the microbiota composition attributable to different biological and technical factors, such as the sample donor, sampling site and device as well as microscopic findings (clinical phenotype). Of the tested variables, microscopic findings (BV-status) explained most of the microbiota variation (53%, p-value 0.0001) followed by bleeding status (19%, p-value 0.0001), the sample donor (11%, p-value 0.0001) and the read count (6%, p-value 0.005). As the read count was significantly associated with the microbiota variation, it was used as offsets in all statistical models. The sampling method explained only 2% (p-value 0.89) of the total variance and hence was practically not associated with the overall microbiota variation. The use of storage buffer (flocked swabs stored dry versus in sterile saline) did not affect the microbiota composition (1%, p-value 0.90).

Despite the high overall similarity of the microbiota profiles between the sampling methods, we quantified their relative distance based on Bray-Curtis dissimilarity using flocked swab, the most commonly employed sampling strategy in the current vaginal microbiota studies [2,40,42,43], as the reference device (Fig C in S1 Appendix). There were no significant differences, however the scape from the lower vaginal 1/3 and cervical brush showed highest dissimilarity to the reference.

The observed microbiota richness ranged from 13 to 47 (median 27) and the diversity from 1.0 to 15.5 (median 2.8) among the samples, being highly variable between the patients (p < 0.05), but essentially invariable between the sampling methods (p > 0.05 for both measures). Because of the small sample size and heterogeneity of the clinical phenotypes (Table A in S1 Appendix), we did not attempt to couple the microbiota composition to subject characteristics.

#### Identification of taxon-specific differences between sampling devices and sites

To identify the individual taxa that potentially differed systemically in abundance between the sampling methods, we compared the bacterial abundance across the sequenced samples. The results indicate that the flocked swab collected less *Atopobium vaginae* than cervical brush and less *Prevotella* cluster 2 than scrape 1/1 (p-values 0.048 and 0.027, fold change 1.03 and 1.28, respectively). Evalyn collected less *Prevotella* cluster 2 than scrape 1/1 (p-value 0.026, fold change 1.40). The cervical brush collected less *A*. *vaginae* and more *Megasphaera paucivorans/sueciensis* than the upper scrape (p-values 0.049 and 0.049, fold change 1.13 and 0.82, respectively) and less *Prevotella* cluster 2 than the scrape 1/1 (p-value 0.028 and fold change 1.33). In all cases the fold changes between the mean abundances of the devices were negligible, maximally 1.4-fold, suggesting that although statistically significant, the biological relevance of the detected species-specific differences is small.

## Discussion

This study provides the first next generation sequencing–based analysis addressing in parallel the impact of different sampling devices and anatomical sites on the vaginal microbiota composition and species diversity. Our results, based on excessively high 16S rRNA gene amplicon sequence coverage of one self-sampled and six different physician-collected samples from 10 women, indicate that both the sampling device and anatomical site have little effect on the representation of the cervico-vaginal bacterial community membership and abundance. In contrast, the amount of the captured total genomic DNA, but not the proportion of bacterial DNA, is strongly dependent on the sampling device and to a lesser amount on the anatomical site of the sampling.

Among the tested sampling strategies, the choice of a sampling tool had the largest impact on the DNA yield from vaginal samples. The self-sampling brush collected a mean of 19 μg of genomic DNA per sample, vastly exceeding the DNA yields obtained with the other tools (mean ranging from 0.6 μg to 9.1 μg), making it therefore ideal for applications requiring a large amount of cells or DNA. We further observed a non-significant, descending trend in the DNA yield from fornix and cervix to vaginal walls (Fig 3B). All scrapes collected invariably low amounts of DNA (Fig 3B). Based on the visual inspection of the cell pellets during DNA extraction, the large surface area of the self-sampling brush collected several times more vaginal content than the other devices, especially the scrapes, and this evidently contributed to the large difference in the DNA yields between the devices. We did not attempt to normalize the DNA concentrations for sample weight but rather calculated the total yield of genomic DNA per each device to reflect the real-life situation. We are not aware of other studies that have compared genomic DNA yield from different types of vaginal samples. Self-collected vaginal Dacron swabs have been reported to yield 2.5 to 5 μg of total DNA [7], which is ca. 2-fold lower than our DNA yields from physician-collected, spatially constrained flocked swabs.

In order to specifically quantify the amount of bacterial DNA in the samples, we quantified the 16S rRNA gene in each sample using qPCR. To normalize for the high device-driven variation in the ab-solute DNA yields, we calculated the results per fixed amount of template DNA (1 ng). While the sampling device had the largest impact on the DNA yield, the amount of bacterial DNA in the samples was driven strongly by inter-individual variation with minor, non-significant differences arising from the sampling methods (Fig 3C). This indicates that each woman had a characteristic bacterial load in the vagina that was robustly collected with all the tested methods. Hence, although self-sampling brush collected significantly more DNA in absolute terms, these samples had similar bacterial content to the scrapes and other samples, highlighting that the majority of the DNA collected by the self-sample is of non-bacterial origin.

We observed a trend towards a lower bacterial content in the cervix compared to the vagina, in line with a previous study [44] that collected vaginal and cervical samples from 100 women and observed higher bacterial load in the vagina measured by qPCR. We also observed somewhat lower amount of bacteria in the lower third compared to the rest of the vagina. However, these differences were minor compared to the biological variation independent of the sampling strategy. In line with an earlier study [45], the BV samples had ca. 10-fold higher total bacterial load compared to other samples. In summary, our results of the quantitative analysis indicate that the vaginal DNA yield depends on the sampling method, but the bacterial counts in the vagina are regulated by biological factors, being insensitive to the choice of the sampling strategy. This also applied to the microbiota diversity and richness that were significantly affected by the sample donor, bleeding and BV-status, but not by the sampling site or device.

Our data indicate that the vaginal microbiota composition assessed by NGS is robust to variation in sampling devices and anatomical sampling sites, as the microbiota profiles and diversity were highly similar between all the tested seven sampling approaches, and sampling strategy explained only 2% of the detected microbiota variation. This study confirms and extents the previous molecular studies reporting the similarity of microbiota profiles derived from self- vs. physician-collected swabs from the mid-vagina [19], and from the physician-collected swabs from the vaginal introitus, the posterior fornix, and the midpoint of the vagina [15]. However, our results disagree with the only previous study that has addressed the impact of vaginal microbiota sampling device and anatomical sampling site in parallel in the same women [46]. The authors reported that both the vaginal sites and sampling methods introduce substantial variation to the detected microbiota profiles. Comparison of the swabs collected from the cervix, fornix and lower 1/3 of the vaginal canal of eight women showed marked, up to phylum-level microbiota differences that however were inconsistent across the study subjects.

The study by Kim et al [46] was the first molecular study to address the niche variation and potential impact of sampling method on the vaginal microbiota. Although comprehensive regarding the number of the tested sampling variables, the work was based on Sanger sequencing of clone libraries and can therefore be considered being shallow according today’s standards for sequencing. Hence, the detected microbiota variability between the samples may at least partly reflect stochastic variation due low sampling depth. Our MiSeq data was saturated in terms of the read counts, and in that sense our results, showing negligible effect of the sampling device on the microbiota profile, can be considered more reliable. Therefore the single swab that has been used to sample the vaginal microbiota in majority of the large-scale studies to date [2,40,42,43] seems to be representative.

When compared to self-sampling methods used in previous studies [18,19,40,47], the Evalyn self-sampling device has two major differences: the external tube to minimize contact with vulva and to keep the sampling depth constant, and the actual sample collecting part being a silicone brush instead of a swab. We did not do any direct comparison to other self-sampling methods, since we did not want to risk losing possible spatial variation by mixing the bacteria across vaginal sites with multiple self-sampling. There are other devices for self-sampling on the market, but we chose Evalyn because of the protective tube, the Finnish-language patient manual available and the fact that we happened to have few extra devices left from another study at our disposal [48].

Based on deep Illumina MiSeq sequencing and validated bioinformatic pipeline for sequence processing, we detected altogether 45 bacterial species in the studied 10 Finnish asymptomatic women. The variation of microbial composition was high between the individuals, six out of 10 women having Lactobacillus-dominated microbiota and the rest had *Enterobacter*, *Lachnospiraceae* BVAB1, *Prevotella* or *Megasphera* dominated community (Fig 4). Four patients had *L*. *iners* dominated (Community State Type III; CST III) and two *L*. *crispatus* dominated bacteria (CST I) [15]. Our small cohort represents the first study to measure the vaginal microbiota with culture-independent methods from Finnish women. Species composition was similar to what has been reported in studies from our neighboring countries Estonia and Sweden [49,50] and in the most recent large meta-analysis of vaginal microbiota [51]. This suggests that our results on the robustness of the microbiota profiles to sampling strategy can be generalized also to healthy women even though many of the 10 women sampled in this study happened to have bacterial vaginosis and/or abnormal cells in Pap smear. The limitation of our study is the relatively small number of study subjects. Hence, we cannot prove that the sampling methods or locations would always produce equal results.

In accordance with the community-wide comparison methods, we detected very few taxon-specific abundance differences between the sampling strategies. The three bacteria (*Atopobium vaginae*, *Megasphaera paucivorans/sueciensis*, *Prevotella* cluster 2) reaching statistical significance differed between cervical brush versus flocked swab, and cervical brush versus upper 1/3 scrape. All the differences had very low fold change in relative abundance (maximally 1.4-fold) and hence the biological relevance of these differences seems virtual. With the targeted 10 patients, we did not identify bacteria that would have been unique e.g. to cervix, or to the adherent material collected from the vaginal wall by the scrapes. Also previously, the microbiota composition between cervical and vaginal samples has been reported to be similar [44]. *G*. *vaginalis*, an important species in the vaginal microbiota, was not covered in our analysis due to the mismatch of the forward primer 27F. Consequently, our study cannot evaluate performance of these sampling methods for *G*. *vaginalis* or any other bacteria belonging to order *Bifidobacteriales* [41]. The parameters used in the preprocessing pipeline filtered low abundant bacteria and thus this study does not address possible variation in the very low abundant species between sampling sites and devices.

In summary, our results indicate that different sampling strategies collect comparable vaginal microbiota composition and diversity. Hence, biological variability of the vaginal microbiota exceeds the extent of technical variation rising from the sampling collection technique (this study) and DNA extraction method [21].Therefore past and future vaginal microbiota studies employing different sampling strategies should be comparable in the absence of other technical confounders, such as sample storage, sequencing strategy or bioinformatic parameters. The Evalyn® self-sampling device performed equally well compared to samples taken by a clinician, and hence offer a good-quality microbiota sample without the need for a gynecological examination. The self-sampling option enables higher participant rates e.g. in large longitudinal epidemiological studies. Our results emphasize the fact that the DNA yield from vaginal samples is merely a qualitative readout, which cannot be used as an indication of bacterial load or microbiota diversity in the samples. However, it might have practical implications for study design, e.g. when selecting a method for other down-stream applications than NGS that require larger input sample volumes, or when aliquoting the samples for multiple assays.

## Conclusion

Vaginal microbiota can be sampled many ways and this study shows that four different devices and six vaginal locations can produce comparable results with almost no differences in the microbiota composition. Ultimately the choice for optimal sampling device depends on the sampling conditions, e.g. if gynecological examination is feasible or not, and on the sampling location of interest. Combined protein and microbiota sampling from one sample should also be possible with a brush or a flocked swab, if multiple sampling is not possible, but a single sample for multiple purposes is very vulnerable and might also lead to multiple freeze-thaw cycles in the laboratory compromising the sample quality.

### Ethical statement

The study was approved by the Ethical Committee of Helsinki University Central Hospital (HUCH, 21/13/03/03/2014) and all participants provided a written informed consent.

## Supporting information

S1 Appendix Additional tables and figures. (PDF)

S2 Appendix R-code. R commands and source code for statistical analyses.
(R) Click here for additional data file.

## Abbreviations

ANOVA: analysis of variance
BV: bacterial vaginosis
BVAB1: BV-associated bacterium 1
CST: community state type
Ct: cycle threshold
DMSO: dimethyl sulfoxide
DNA: deoxyribonucleic acid
EDTA: ethylenediaminetetraacetic acid
FDR: false detection rate
HPV: human papilloma virus
HUCH: Helsinki University Central Hospital
NGS: next generation sequencing
OTU: operational taxonomic unit
PBS: phosphate-buffered saline
PCoA: principal coordinates analysis
PCR: polymerase chain reaction
qPCR: quantitative PCR
RBB: repeated bead beating
rpm: rotations per minute
rRNA: ribosomal ribonucleic acid
SDS: sodium dodecyl sulfate
