# Supplementary material for: Comparative analysis of vaginal microbiota sampling using 16S rRNA gene analysis
Source: PLoS One. 2017 Jul 19;12(7):e0181477. doi: 10.1371/journal.pone.0181477 (PMC5517051; doi:10.1371/journal.pone.0181477)
Supplement: S1 Appendix — (PDF) [file pone.0181477.s001.pdf]

S1 Appendix

Additional tables and figures for comparative analysis of vaginal microbiota sampling using 16S rRNA gene analysis, Virtanen et al.

Table A. **Study population.** Patient 1. had taken oral cephalexin one month before sampling. Abbreviations: LSIL: low-grade squamous intraepithelial lesion; HSIL: high-grade squamous intraepithelial lesion; CIN1-3: cervical intraepithelial neoplasia, grade 13; ASC-US: atypical squamous cells of undefined significance; ASC-H: atypical squamous cells - cannot exclude HSIL; LEEP: loop electrosurgical excision procedure.

Patient	Age	Referral indication	Colposcopy Pap smear	Biopsy	Microscopy	Contraceptive
1	27	Other	Normal	Normal	Atrofia post partum	Breastfeeding/condom
2	30	LSILx2	Normal	Normal	Mixed bacteria	Harmonet®
3	39	HPV+ x3	Normal	CIN2	BV	Mirena®
4	45	HSIL	HSIL	CIN2	Inflammation	Mirena®
5	33	CIN1 LEEP	ASC-US	Normal	BV	none
6	42	ASC-H/HSIL	LSIL	Atypia coilocytaria	BV and fungus	Progesterone (undefined)
7	32	LSILx2	ASC-US	Inflammation	Normal	none
8	46	LSIL	LSIL	CIN3	BV	Sterilization
9	32	LSIL	Normal	-	Mixed bacteria	Pills (undefined)
10	48	LSIL	LSIL	Inflammation	Mixed bacteria	Sterilization

Table B. **Sequenced samples**. List of samples chosen for sequencing by devices and patients.

#	Sampling Device	Number of Samples Sequenced	Patients Excluded
1	Evalyn	10	
2	Flock right fornix	10	
3	Flock left fornix	5	5,7,8,9,10
4	Lower 1/3 right	5	5,7,8,9,10
5	Upper 1/3 right	10	
6	Scrape 1/1 left	10	
7	Cervical brush	10	

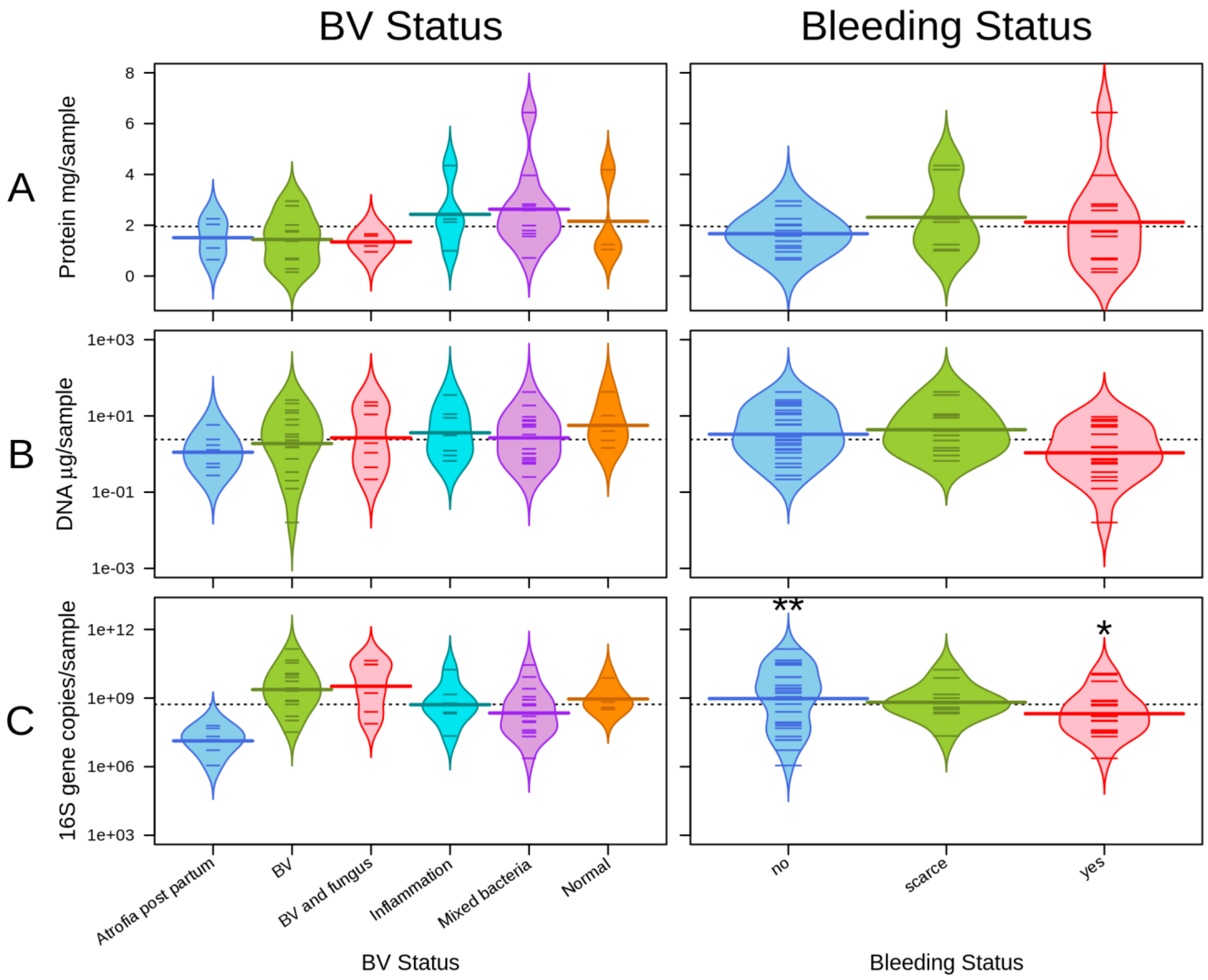

Fig A. Protein and DNA results compared to BV- and bleeding status. Protein yield (row A), DNA yield (row B) and total bacteria measured by qPCR (row C) per bacterial vaginosis (BV) status (left) and bleeding status (right). The mean values not sharing the same number of asterisks signify samples with statistically significant ($p < 0.05$) pair-wise differences.

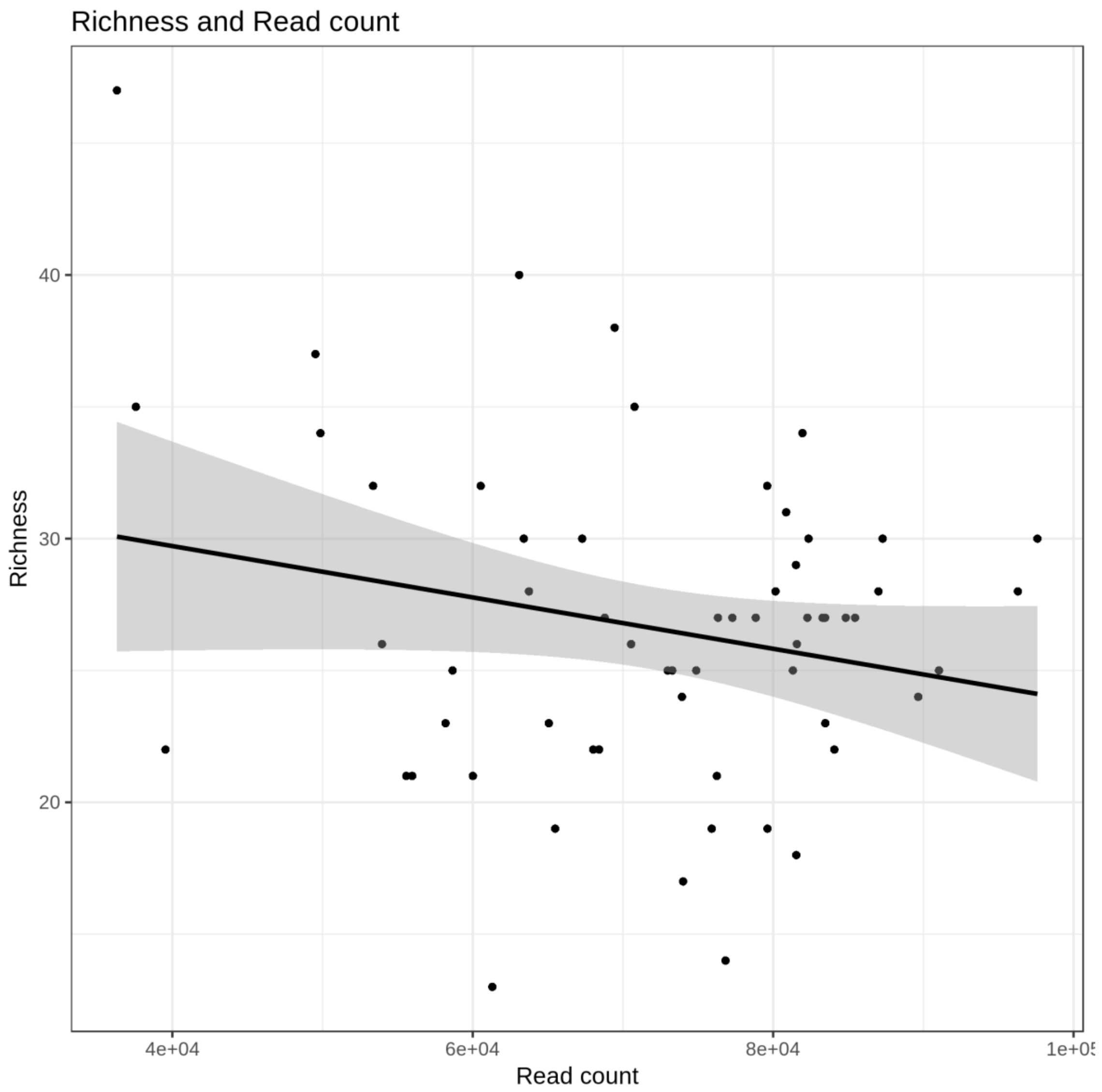

Fig B. **Richness and read count comparison.** Measured richness compared to read count in samples. The black line indicates linear fit with shaded 95% confidence region.

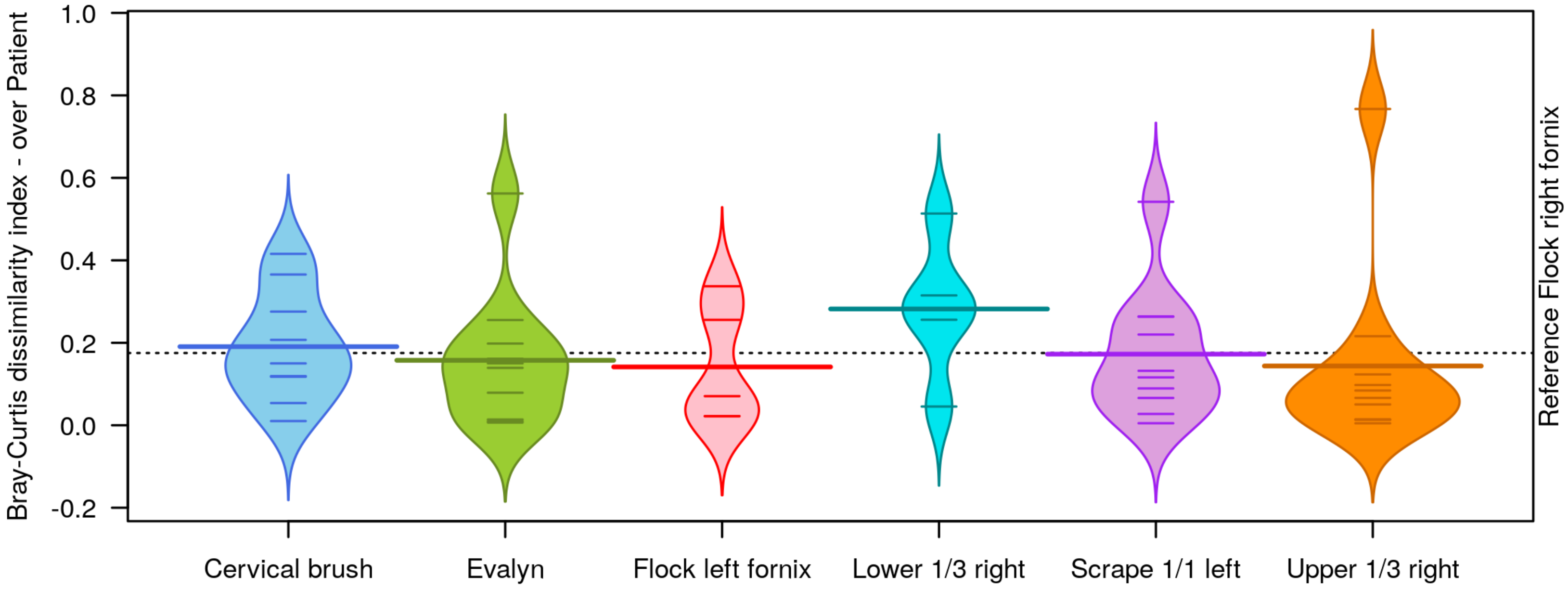

Fig C. Dissimilarity cmparison between sampling devices and -locations. Bray-Curtis dissimilarity of the vaginal microbiota samples, calculated for all the sampling methods using the flock swab sample from the right fornix as a reference. No statistically significant differences were detected.
